# Visualising phase change in a brushite-based calcium phosphate ceramic

**DOI:** 10.1038/srep32671

**Published:** 2016-09-08

**Authors:** A. Bannerman, R. L. Williams, S. C. Cox, L. M. Grover

**Affiliations:** 1 School of Chemical Engineering, University of Birmingham, B15 2TT, UK

## Abstract

The resorption of brushite-based bone cements has been shown to be highly unpredictable, with strong dependence on a number of conditions. One of the major factors is phase transformation, with change to more stable phases such as hydroxyapatite affecting the rate of resorption. Despite its importance, the analysis of phase transformation has been largely undertaken using methods that only detect crystalline composition and give no information on the spatial distribution of the phases. In this study confocal Raman microscopy was used to map cross-sections of brushite cylinders aged in Phosphate Buffered Saline, Foetal Bovine Serum, Dulbecco’s – Minimum Essential Medium (with and without serum). Image maps showed the importance of ageing medium on the phase composition throughout the ceramic structure. When aged without serum, there was dissolution of the brushite phase concomitant to the deposition of octacalcium phosphate (OCP) around the periphery of the sample. The deposition of OCP was detectable within five days and reduced the rate of brushite dissolution from the material. The use of serum, even at a concentration of 10vol% prevented phase transformation. This paper demonstrates the value of confocal Raman microscopy in monitoring phase change in biocements; it also demonstrates the problems with assessing material degradation in non-serum containing media.

Calcium phosphate (CaP) cements have attracted significant interest as bone graft replacements due to their ability to facilitate bone growth across their surface *in vivo*^1^,^2^. As they are ceramic however, they are brittle, which can lead to a long-term risk of failure if they are not resorbed and replaced by new bone. Consequently, there has been growing interest in the development of sparingly soluble cements that have a high resorption rate since they are not only resorbed through osteoclastic action, but also through dissolution. Brushite (CaHPO_4_.2H_2_O) is one such material that exhibits a solubility orders of magnitude higher than hydroxyapatite (Ca_10_(PO_4_)_6_(OH)_2_)^3^. A number of studies have investigated the degradation and biological resorption of brushite cements under both *in-vitro* and *in-vivo* conditions. These studies have shown brushite dissolution to be unpredictable with implants having been shown to remain stable^4^, undergo fragmentation^5^, or be resorbed^6^,^7^,^8^. Degradation is still poorly understood, but research has shown a close relationship between local conditions and cement composition. Additionally the phase composition and structure has been shown to play an important role in bone development^9^,^10^. Enhancing the understanding of the chemical and biological mechanisms involved in resorption is essential to optimising the cement for successful long-term bone regeneration.

Analysis of the temporal changes in brushite and other resorbable samples has largely focused on changes in crystalline composition studied through high resolution topological imaging such as scanning electron microscopy, coupled with bulk compositional properties obtained via spectroscopy of powdered samples^11^,^12^,^13^. This provides high-resolution information of the porosity and crystal structure, but gives poor to no spatial resolution of the chemical constituents in a system where the structure and environment cannot be considered as homogeneous. Further to this, the reliance on crystalline analysis limits the information that can be gained, with X-ray diffraction only able to detect >5 wt% of a material. This is a major flaw when it is known that extensive re-precipitation slows degradation and previous work has shown amorphous phases have been detected early during ageing in some cases^14^. Radiographs and histology^4^,^5^,^6^,^7^ can provide further information on compositional changes during resorption in *in-vivo* implanted samples, but are limited in the level of chemical and biological information they can provide. The geometrical distribution of phases may be of particular importance where a coating in a less soluble phase, in physiological conditions will impede dissolution of the other phases.

In order to detect smaller quantities of a material, regardless of crystallinity and provide geometrical information on these phases, it may be possible to utilise a chemical imaging method such as confocal Raman microscopy (CRM). The use of Raman spectroscopy in the analysis of brushite is well established with phases and their vibrational modes assigned to Raman peaks^15^, and numerous studies applying the technique as a means to probe the composition of samples have been reported^16^,^17^,^18^,^19^. CRM extends the scope of Raman spectroscopy by taking individual spectra at a number of locations it allows intensity maps of peaks to be plotted showing the intensity and distribution of each phase to a high resolution - up to 200 nm - over a single plane^20^.

Despite the advantages offered, the number of image mapping based Raman studies in the field is limited with only a small number of studies looking at or relating to the ageing of calcium phosphate cement implants known to the authors^21^,^22^,^23^,^24^. In part, the low level of use in the literature has been due to technological limitations with long spectral acquisition times, giving poor data quality. With technological improvements in recent years and a range of related techniques that provide a stronger signal allowing much shorter acquisition times.

This study reports the use of CRM to evaluate the phase changes that occur during the ageing of brushite based calcium phosphate cements. In doing this, it is now possible to gain an unprecedented insight into the chemical changes that occur within this material during ageing. CRM was utilised to map the evolution of chemical changes in brushite CaP cement cylinders aged in different media; phosphate buffered saline (PBS), foetal bovine serum (FBS), and Dulbecco’s modified eagle media (DMEM) without or supplemented with FBS. Comparing the mapping results to bulk spectra of the samples acquired through X-ray diffraction (XRD) and Raman point spectroscopy verified the results and highlights the advantages of CRM.

## Materials and Methods

### Cylinder production

To produce brushite cement cylinders β –tricalcium phosphate (Ca_3_(PO_4_)_2_; β-TCP) (Plasma-Biotal, Tideswell, Derbyshire, UK) was mixed with 3.5 M orthophosphoric acid (Sigma-Aldrich, Gillingham, UK) at a powder to liquid ratio of 1.75 g/ml. The mixture was poured into custom-made moulds to produce 12 mm (height) by 6 mm (diameter) specimens, and left to set overnight.

### Cement ageing protocol

Cement cylinders were sterilised under ultraviolet light overnight. Sterilised cylinders were placed into 50 ml centrifuge tubes with 20 ml of either DMEM (Sigma-Aldrich, Gillingham, UK), supplemented DMEM as used in cell culture (supplemented with 10% FBS (Sigma-Aldrich, Gillingham, UK), 2.4% HEPES buffer (Sigma-Aldrich, Gillingham, UK)), FBS, or sterile PBS (Oxoid, Basingstoke, UK). The ratio of media volume (mL) to solid volume of cement (cm^3^) used in this study was approximately 60:1 (20 mL media to 0.33 cm^3^ cement). Samples were incubated at 37 °C for 10 days in the FBS and DMEM solutions, and for 5, 10, 20, 30 and 50 in PBS. Media was changed daily in each case. pH readings were taken from day 1 to 30 in the PBS aged cylinders. Cylinders were removed from ageing medium, washed in distilled water, dried overnight at 37 °C, and then cut using a scalpel across the midpoint to expose the cross-section. Individual halves were mounted on a sample holder using distilled water and frozen at −20 °C in a cryostat (Starlet 2212, Bright, Cambridge, UK), samples were cryotomed in 2 μm steps to produce a smooth surface suitable for confocal imaging. They were then briefly washed with distilled water to remove any debris, and dried at 37 °C before imaging.

### Raman mapping

Mapping was performed using a confocal Raman microscope (Alpha300r, Witec, Ulm Germany) with a 785 nm 300 mW argon laser (Toptica photonics, Munich, Germany). Spectra for DMEM, FBS and day 10 PBS samples were collected between 0–3000 cm^−1^ with a mean spectral resolution of 3.6 cm^−1^, using a 300 g/mm with 750 nm blazing grating spectrograph (Acton SP2300, Princeton instruments, Trenton, NJ, USA). For the extended PBS media ageing, a 60 mW 514 nm argon laser (Spectra-Physics, Santa Clara, California, USA) was used, with data collected over a spectral range of 200–1200 cm^−1^ with a mean spectral resolution of 1.2 cm^−1^, using a 1800 g/mm with 500 nm blazing grating (Acton SP2300, Princeton instruments, Trenton, New Jersey, USA). Mapping was collected for whole area scans using a 5 × 0.1 NA atmospheric lens (Nikon, Tokyo, Japan), over a 6500 × 6500 μm area with 100 × 100 points collected with an integration period of 1 s. Higher resolution scans at the cross section edge were acquired with a 20 × 0.45 NA atmospheric lens (Nikon, Tokyo, Japan), over an area of 1000 × 1000 μm, with 100 × 100 points collected and 1 s integration time. Laser spot sizes were calculated for the 100 × lens (λ = 514 nm D_xy_ 391 nm D_z_900 nm, and λ = 785 nm D_xy_598 nm D_z_126 nm), and for the 20 × lens (λ = 514 nm D_xy_ 696 nm D_z_2538 nm and λ = 785 nm D_xy_1064 D_z_3876). Acquisition of single spectra from points of interest was performed by taking 20 accumulations of three-second integration periods. To prevent movement during scanning, samples were attached to a 35 mm well plate using double-sided tape.

### Processing Raman data

Data was pre-processed to remove cosmic rays using the instrument associated software (Witec Project v2.10. Witec, Ulm, Germany), exported into MATLAB (MATLAB 2011a. Mathworks, Natick, Massachusetts, USA), baseline corrected using a least squares penalised method^25^, and vector normalised. Least squares regression was used for fitting of individual Gaussians to each peak. False colour image maps were produced by integrating over the sum of peaks of interest. For further processing the dataset was segmented to separate the sample from background and allow calculation of the measured area and centroid. The dominant phase in each pixel location was determined by the association of the peak with the highest intensity. Measurement of OCP penetration distance at each time point was performed using ImageJ (ImageJ v1.43u. National Institutes of Health, Bethesda, Maryland, USA).

### XRD and Raman bulk spectra

Samples from each PBS time point were dried and then ground using a mortar and pestle. Raman spectra were acquired at five randomly chosen points from each powder sample with 20 accumulations at three second integration time per accumulation. Resulting spectra were pre-processed to remove cosmic rays and Savitzky–Golay filtering was applied using the associated Witec Project software and averaged together. Average spectra were exported and base-line corrected in MATLAB. For XRD, approximately 500 mg of the powder was distributed over a 10 mm diameter circular area of Scotch tape and attached to the sample holder. XRD patterns were acquired using a Bruker D8 Advance Diffractometer (ASX Gmbh. Bruker, Karlsruhe, Germany) using the copper K-alpha line 1.5406 nm at 40 kV and 30 mA. Diffraction data was collected between 2-θ = 5–60° at a rate of 0.05°/step. Samples were rotated about its axis during the entire measurement to minimise the influence of preferential crystal orientation. Data was baseline corrected, and smoothed using MATLAB. Peak identification was performed using International Centre for Diffraction Data cards 00-009-0169 (β-TCP), 04-013-3344 (brushite), and 00-044-0778 (OCP).

## Results

### Brushite cement ageing

A 60 mol% maximum conversion of β-TCP to brushite was expected based on the ratio of precursor materials used, which react according to equation 1.


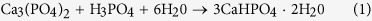


Cylinders were aged in a dynamic solution of either: FBS, DMEM, DMEM supplemented with 10% FBS, or PBS for 10 days. A longer study of PBS aged samples was performed with PBS over 50 days. In each case aged samples showed no visible signs of degradation compared to non-aged. pH readings taken for the first 30 days of ageing in PBS showed an initial decrease from pH 7.3 to pH 6.5. After which a steady return toward the baseline pH of PBS (7.3) was observed.

### Confocal Raman Mapping of samples aged in different media

**Figure 1:**
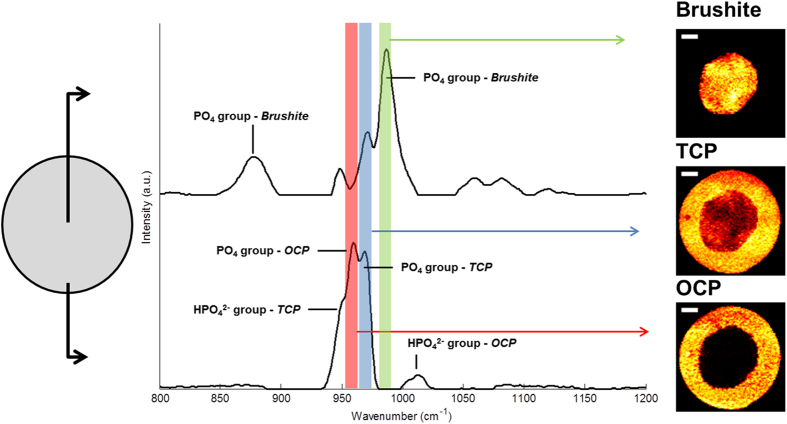
Raman image formation from a day 50 sample showing spectra taking from the centre and outside of the cross section, showing the relative intensities of the main peaks associated with each phase. Each peak was mapped to the specimen showing the distribution of brushite, TCP and OCP over the cross section. Scale bar 1000 μm.

CRM mapping was performed on cylinders that were aged for 10 days in each media using a 785 nm laser. Calcium phosphate phases were identified based on Raman spectral peak locations previously reported in the literature^15^ (Table 1.). The P-O stretching mode (*v*1) of the PO_4_ group which has a unique peak shift for brushite (985 cm^−1^), octacalcium phosphate (OCP, Ca_8_H_2_(PO_4_)_6_.5H_2_O) (958 cm^−1^), and TCP (970 cm^−1^) was used as the main reference peak for each CaP phase. Spectra at the centre and edge of a cylinder aged for 50 days in PBS are shown in Fig. 1. A −5 cm^−1^ shift in the peak location was observed for samples aged in DMEM and FBS based media. Prior to image formation datasets were filtered to remove cosmic rays, baseline corrected, and normalised. Due to the close proximity of the peaks (~12 cm^−1^) relative to the spectral resolution of the grating (~3.5 cm^−1^) and their FWHM (~10 cm^−1^), a significant overlap was present which would give rise to misleading results as constructive interference increased the apparent intensity of weaker or non-present peaks. A classical least squares regression was performed to fit an individual Gaussian to each peak in the region for image mapping calculations to be taken from. It should be noted that intensity values were produced to be consistent across phases for each sample mapping but not between every data set.

**Figure 2:**
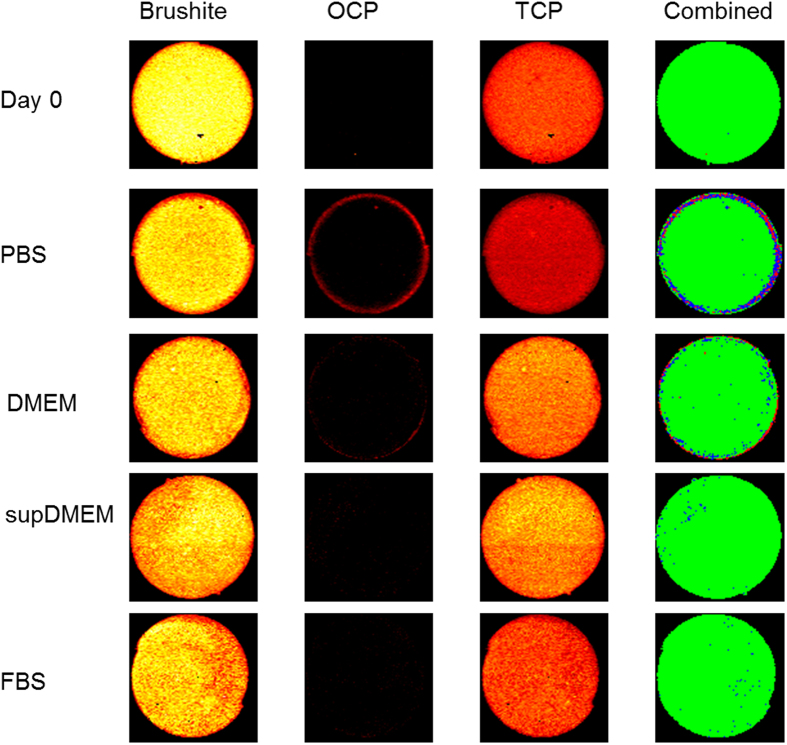
Confocal Raman microscopy heat maps of cylinder cross-sections after 10 days ageing in dynamic media. Heat mapping of the PO_4_ brushite, OCP and TCP phases for cylinders aged in each media condition. The combined images show the dominant phase at each location determined by highest peak intensity; green - brushite, red - OCP, blue - TCP. Scale bar 1000 μm.

**Figure 3:**
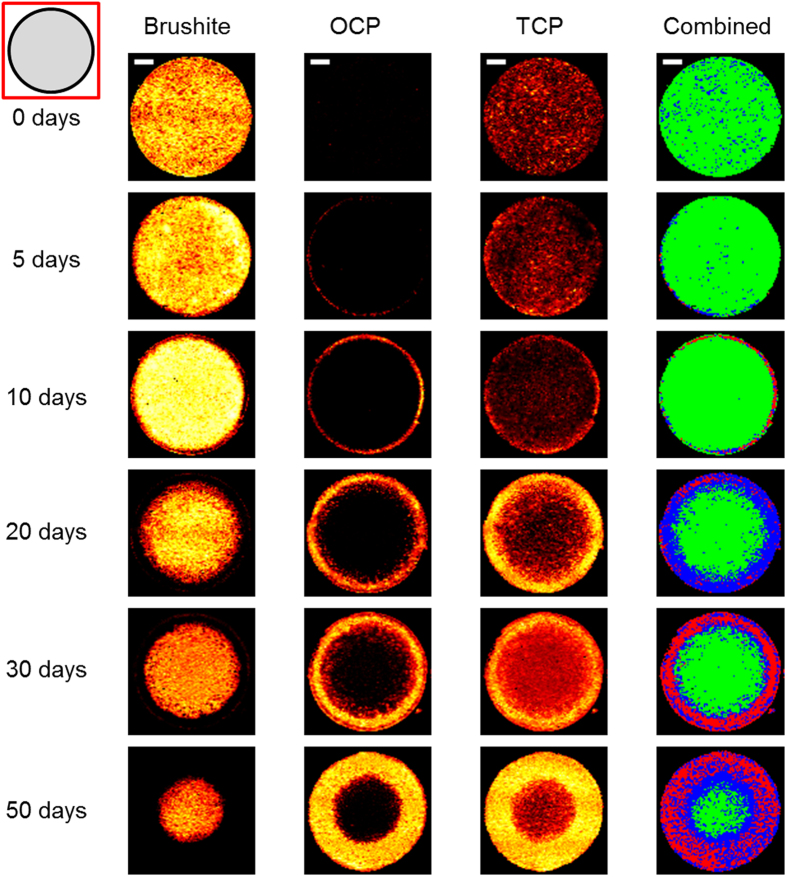
Confocal Raman microscopy heat maps of cylinder cross sections after 0, 5, 10, 20 and 30 days in dynamic PBS showing the intensity mapping of the brushite, OCP and TCP phases at each time point. The combined image shows the dominant phase at each location; green - brushite, red - OCP, blue - TCP. Scale bar 1000 μm.

Image mapping of samples that were aged for 10 days in media for whole cross sections (Fig. 2) and edge sections (Fig. 3) showed clear differences in the cement composition dependent on the media content. In un-aged samples a homogeneous structure was present with the most intense peak being that indicative of brushite, and lower more disperse peaks indicative of TCP. OCP was not detectable beyond the background noise. At 10 days a shell of OCP was observed around the outside of the cylinder in the PBS and non-supplemented DMEM samples. A higher quantity of brushite dissolution and deeper penetration of OCP was apparent in the PBS aged sample, whereas the DMEM aged sample had OCP form largely on the outside of the cylinder itself. Conversely the supplemented DMEM and FBS samples, with 10% and 100% serum respectively, showed no evidence of brushite dissolution or OCP formation.

Image mapping data acquired was processed to provide further visualisation data on the phase quantity and distribution. Mappings were segmented, with the dominant phase at each spatial location determined based on maximum peak intensity. This was used to produce composite images showing the dominance of the phases at each time-point for the whole area (Fig. 2) and edge (Fig. 3). Mapping of the dominant phase at each spatial location corroborated the heat map images for each peak, showing a solid ring of OCP around the outer edge of PBS and DMEM aged samples, with a higher density of TCP forming around and to a greater penetration depth than the OCP. Analysis of the Raman spectra showed no evidence of hydroxyapatite formation in any sample.

### Confocal Raman Mapping of samples aged in PBS

**Figure 4:**
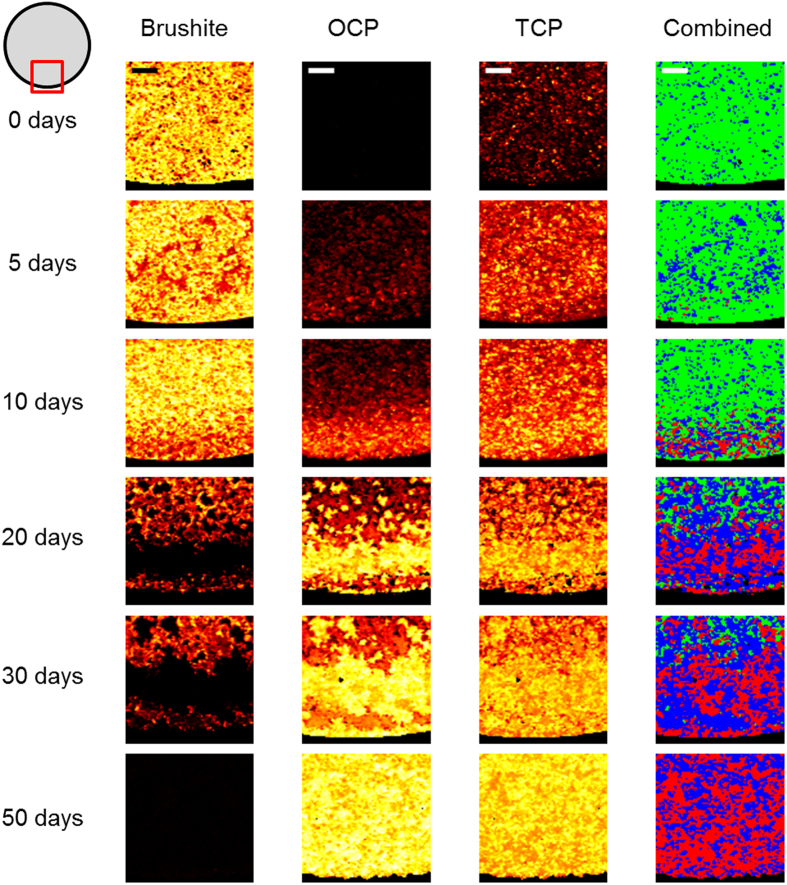
Confocal Raman microscopy heat maps of the outer edge of cylinder cross sections after 0, 5, 10, 20, 30 and 50 days in dynamic PBS showing the intensity mapping of the brushite, OCP and TCP phases at each time point. The combined image shows the dominant phase at each location; green - brushite, red - OCP, blue - TCP. Scale bar 200 μm.

**Figure 5:**
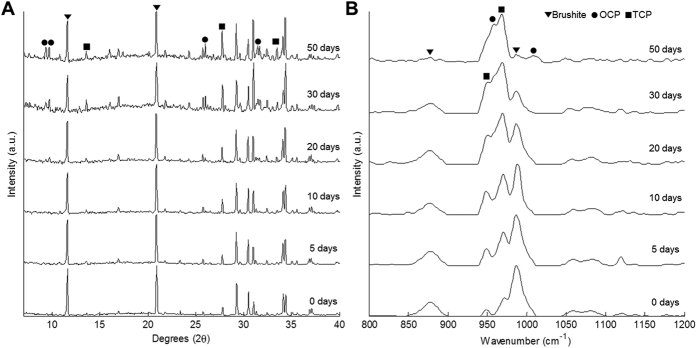
Bulk compositional analysis of powdered samples at each time point from the PBS ageing series acquired using (**A**) XRD and (**B**) Raman spectroscopy.

Having found that CRM mapping of brushite cylinders was able to spatially determine phase change in media with different compositions, the study was extended to encompass a longer time series allowing a deeper investigation of the material evolution. Raman imaging mapping of cylinders dynamically aged in PBS for 50 days was performed and processed as before over the whole cylinder cross section (Fig. 4) and at a higher resolution over the edge region (Fig. 5). A 514 nm laser was used due to the minimal auto-fluorescence in PBS aged samples, enabling a higher signal intensity and access to a higher spectral resolution grating for improved differentiation of the PO_4_ peaks.

Image mapping over the time series showed results similar to those from the PBS and non-supplemented DMEM seen in Figs 2 and 3. An initial largely uniform phase distribution dominated by brushite, with a weak TCP signal, and no OCP was seen for the un-aged cylinder. At five days, a layer of OCP was observed at the outside of the cylinder, with a higher density of TCP both about the OCP and penetrating further into the sample. At each subsequent time point the layer of OCP and TCP increased in area and radial penetration. Further analysis of the spectra supported the results shown by the PO_4_ peaks. With the decay of the 878 cm^−1^ PO_4_ peak associated with brushite alongside the drop in intensity and complete removal of the 985 cm^−1^ peak at the outside of the cylinder, and the appearance of the 1010 cm^−1^ P-O HPO_4_^2−^ OCP peak alongside the main PO_4_ OCP peak.

From these results it can be determined that over time in dynamic PBS the outer layer of brushite in the cylinder was eroded away as it was dissolved, leaving a TCP structure which was replaced by OCP. The rate at which the dissolution of brushite and precipitation of OCP occurred for a particular location was clearly shown to be dependent on the distance from the cylinder edge, with initial OCP appearance by five days at the edge of the cylinder, the outer radius of brushite being completely replaced by 10 days, and only a small central core of brushite remaining at 50 days. As seen in the PBS results (Fig. 3) a thin (approximately 50–200 μm) layer of brushite was observed on the outside of the cylinder at aged time points - notably at day 20 and 30 in Fig. 5 - despite the otherwise complete dissolution of brushite 100 μm or further into the sample. This layer was seen to degrade and be replaced by OCP after 50 days. Composite images of the three phases showing the dominant phase at each point showed the emergence of a distinct three phase structure with phases clustered by radial distance as the brushite underwent dissolution, leaving the TCP scaffold which was subsequently seeded by OCP. The percentage of the total area for each phase was calculated for each time point based on these results (Table 2). In addition an estimate of the penetration distance of OCP was determined using composite images (Table 2). Both of these supported the visual observations of brushite dissolution and OCP penetration. Such quantifications were complicated by random variation of TCP distributions between samples and the assumption of the most intense peak being sufficiently dominant, but allow a reasonable estimate and insight of the phase composition at each time point and its evolution.

### Bulk compositional data

Bulk compositional data from powdered samples was acquired using XRD and Raman spectroscopy at each time point for the extended PBS ageing series. The XRD time series (Fig. 5(A)) showed the intensity of peaks associated with brushite decrease compared to those related to TCP, with a subsequent appearance and rise in OCP associated peaks over the 50 day time period, matching the general CRM results. Likewise the Raman spectra time series (Fig. 5(B)) showed clear evidence of a decrease in the 987 cm^−1^ PO_4_ brushite peak from initially the strongest, to almost dropping beyond the background noise at day 50. Whilst the OCP and TCP associated peaks increased in intensity becoming the dominant phases. In both the XRD patterns and Raman spectra the presence of OCP was not detected beyond the background at the day 5 or day 10 time points despite the presence being observed in the CRM image mapping results. This was most likely due to the relatively small quantities of OCP at those time points being beyond the sensitivity of the instruments. As before none of the major peaks associated with hydroxyapatite were present, with any minor ones in the XRD clashing with those from the major phases found in the sample. Suggesting that hydroxyapatite was not present in any detectable quantity.

## Discussion

This study investigated the visualisation of phase changes using Raman imaging on brushite cement cylinders aged in a range of media. Importantly it was observed that in PBS aged samples a shell of OCP formed but did not prevent further dissolution, with continued loss of brushite and penetration of OCP over the entire time course. It was also shown that the presence of protein inhibited phase change, even at serum concentrations as low as 10vol%.

The transformation from brushite to a more stable phase such as hydroxyapatite has been shown to be an important factor in the highly unpredictable degradation of brushite cements under physiological conditions^26^. Ageing of brushite cements *in-vivo*^4^,^5^,^6^,^7^,^18^,^26^ and *in-vitro*^16^,^23^,^27^,^28^ has been undertaken by a number of groups with inconsistent results. For *in-vitro* ageing a range of ageing mediums including distilled water^29^, simulated body fluid (SBF)^23^, PBS^16^,^23^,^27^, cell culture medium^17^,^30^, and bovine serum^16^ have been used. In these studies brushite has been shown to undergo dissolution and transformation to OCP, as reported here, or hydroxyapatite depending on the cement composition and conditions it has been aged in. OCP is known to be metastable and will transform to hydroxyapatite^3^, however this was not observed in the time frame of this study. As a result of these variations in phase transformation, amongst other differences in degradation, there is an ongoing debate as to which media composition most accurately duplicates native physiological conditions^30^,^31^.

As discussed earlier, much of the analysis performed in studies on the physiological ageing of CaPs has focused on a combination of morphological imaging coupled with bulk spectral compositional properties. Producing information on the overall composition and properties such as crystal size but severely limiting the depth and scope of phase information available. Some studies have used methods acquiring spectral information at a number of points; providing information on changes in calcium to phosphate ratios^26^ and phase as a function of spatial location^18^,^26^. These studies, however, are still limited, providing a reduced view with susceptibility to impurities or variations in compositional structure, or conversely to miss those elements. Raman imaging presents a means to provide more detailed compositional information on the evolution of phase composition, being able to map molecules and species without the need to label or destructively prepare samples. Whilst Raman spectroscopy is unable to provide quantitative information on the quantity of each material present, it is able to identify and provide information on the species present and their relative intensities. Previously we have applied Raman mapping to the interface in a brushite-hydrogel system; showing evidence of the cement undergoing dissolution or fragmentation, and transforming into OCP when aged in a PBS or SBF media^23^. The work presented in the current study gives a more controlled experimental environment allowing a clearer visualisation of the phase changes occurring as the samples aged.

For cylinders aged in PBS a three-phase structure was seen to emerge and continuously develop as brushite underwent dissolution, leaving a scaffold of the original TCP reactants, which was subsequently surrounded by OCP. This suggested a non-direct route of conversion from brushite to OCP given the buffer region dominated by TCP between the two phases. The difference in intensity, however, may also be due to different Raman sensitivity inherent with each of the phases. The presence of such a buffer region at the interface between the two phases could be due to brushite being partially hydrolysed to an amorphous calcium phosphate (ACP) phase, before reacting with ions from solution to form OCP. The conversion of ACP to OCP has been reported previously^22^,^32^,^33^. However, the characterisation of ACP by Raman spectroscopy is problematic due to the non-crystalline nature of the species resulting in very broad peaks^34^. This is further complicated by the presence of up to two or three other phases at any given spatial point in the buffer region, with largely overlapping spectra in the region of interest. The main vibrational peak indicative of ACP is given as a broad PO_4_ shifted to 950 cm^−1^ 32 with a high width for all other associated peaks. Analysis of the data failed to find sufficient evidence of the presence of such a peak in the buffer region. Comparison of the ratio of peak intensity at 970 cm^−1^ to 950 cm^−1^ to look for variations which might be indicative of a ACP PO_4_ shift contributing to the overall signal of the HPO_4_^3−^ TCP peak about 950 cm^−1^ found no significant variation across the buffer region compared to any other areas regardless of time point. Given the highly soluble nature of ACP at more acidic pH a high rate of conversion to OCP would be expected^35^ leaving only a small quantity at any given time point. This may be explained by the formation of more stable ACP phase as a precursor to OCP as described by Christoffersen *et al.*^33^ or the effect of concentration gradients in the porous structure and kinetic stabilisation reducing the conversion rate. Regardless of the conversion mechanism, the outside shell of OCP was not seen to prevent brushite dissolution further into the sample.

The transformation of brushite to OCP itself is surprising given the lower stability of OCP compared to hydroxyapatite predicted in solubility isotherms, with the majority of cases in the literature reporting the transformation of brushite to hydroxyapatite when aged in a neutral or slightly alkaline media. However, whilst isotherms allow a prediction of the thermodynamically preferred phase, the kinetics of conversion in supersaturated media can mean that a less stable but more kinetically favourable phase grows faster and defines the final structure. Hydrolysis of brushite to OCP in a pH environment of 6.2–7.4 between 25 °C and 37 °C due to much higher conversion rate to OCP over hydroxyapatite was reported by Tung and Brown^36^. Importantly the brushite cements used in this study were not formed with a setting retardant or calcification inhibitor unlike many others reported studies. In a similar circumstance Malsy *et al.*^37^ reported brushite converted to OCP and apatite in magnesium free cement mixtures. The direct transformation of brushite to OCP is given by Mert *et al.*^38^ as one of the following reactions:


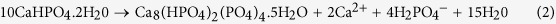



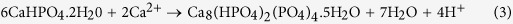



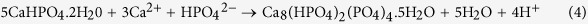


Such a direct route would explain the decrease in pH from 7.3 to 6.5 as the brushite was first exposed to PBS resulting in a high level of dissolution, with the pH subsequently rising toward 7.3 as the remaining quantity of brushite decreased and became more deeply embedded inside the cylinder reducing the dissolution rate.

The heat maps in Figs 4 and 5 suggests an increase in TCP alongside the appearance of OCP, however this is a misleading effect due to the loss of brushite in areas where dissolution had taken place. TCP, which is less soluble than brushite^39^, has a higher intensity signal in these positions compared with areas where brushite has not undergone dissolution. Formation of further TCP (or whitlockite) is unlikely without stabilisation from magnesium ions^39^, which were not present in the cylinder or PBS media.

The variation in initial OCP seeding location between the PBS and basic DMEM media in Fig. 3 suggested a difference in the precipitation routes. In DMEM aged samples, the majority of OCP formed on the outside of the cylinder, whilst in the PBS aged samples the outside remained dominated by a thick band of brushite even after 30 days, with brushite further into the sample undergoing dissolution and being replaced by OCP. The formation of OCP on the outside of the cylinder may be caused by adsorption of components in the DMEM media on the cylinder surface enabling OCP precipitation and possibly limiting, but not preventing, transport in and out of the structure. The presence of a less soluble outer band of brushite as seen in the PBS aged samples raises the issue of uniformity in the cement as a result of local conditions during setting, which may have significant consequences for *in-vivo* implants. In such situations phase composition will determine local pH and have a marked effect on the ingrowth of cells on implanted cements. The stalling or reduced rate of cell migration could cause complications if conversion of brushite to a more stable phase outpaces resorption, and/or regions of highly insoluble cement are left after otherwise total resorption.

The results of this study have shown that the evolution of multiple CaP phases can be mapped based on their Raman signal intensity distribution over a series of time points. Differences in phase transformation based on media were observed, matching previous reports that showed the presence of proteins in serum based media inhibiting dissolution^16^. Previous ageing work on similar samples has noted morphological changes around the outside of aged brushite cylinders under scanning electron microscopy^27^ coinciding with detected overall sample degradation and mechanical changes. This matches the changes seen in the PBS ageing series as the outer ring of OCP was seen to emerge. The ability to accurately resolve and display spectral information at a number of points across the sample adds a considerable contribution to the understanding of the cement and its bioactivity. High resolution imaging of phases will enable a better understanding of the rate and consistency of phase conversion. This is especially significant as we have shown that the acquisition of spectra through bulk methods gives a much less sensitive result, with the emergence of phases in small quantities not clearly detected until a number of days after they were observed under CRM. Furthermore, the compositional information that can be obtained from the Raman spectra is far greater than can be obtained through the histological staining reported in the literature, and does not require lengthy and complex sample preparation.

The major failing of the presented methodology is that it is mildly destructive, requiring physical preparation of the sample to access the plane of interest and make it suitable for confocal imaging. The depth penetration of confocal microscopy in turbid media is at most 200 μm which will be much lower in cements. This limits the application of CRM to *in-vitro* and *ex-vivo* processed samples. However, recent development of techniques for deep Raman data collection in soft tissue and bone^40^ may make non-invasive *in-vivo* imaging of cements to depths in the order of tens of millimetres plausible. Raman is also diffraction limited in its resolution to a maximum of about 200 nm, which will be reduced if a low numerical aperture lens is used to view a larger area, and/or a longer wavelength laser is used to reduce fluorescence and chance of damage to the sample. Depending on the cement system in question the resolution will likely be equivalent to or much greater than the size of individual crystals meaning that it may be unable to resolve to the level of individual crystals with each spectra being composed of a combination of multiple phases, as seen in this study. Due to the highly complex nature of brushite cements and the number of variables that affect the cement bioactivity degradation the use of multiple complimentary techniques is of course still required, though the application of chemical imaging adds a powerful tool.

## Conclusion

We have shown that localised information on chemical composition and phase changes in brushite CaP cements can be mapped and quantified using CRM. This enabled monitoring of the simultaneous evolution of brushite, TCP and OCP phases in samples aged *in-vitro*. Giving clear information on the distribution and relative quantity of each phase over time and allowing a deeper insight and study of the mechanisms involved in the transformation between phases. This chemical mapping provides an important information bridge between the morphology based high-resolution electron microscopy imaging, and bulk chemical composition results often applied in the literature. Applied to further *in-vivo* and *in-vitro* results such information will enable a deeper level of understanding into the reaction of the sample to the environment and the biochemical mechanisms involved, allowing for the improved optimisation of brushite cements for bone graft applications.

## Additional Information

**How to cite this article**: Bannerman, A. *et al.* Visualising phase change in a brushite-based calcium phosphate ceramic. *Sci. Rep.* **6**, 32671; doi: 10.1038/srep32671 (2016).

## Figures and Tables

**Table 1:** Major Raman peaks found in the 800–1050 cm^−1^ region of interest and their phase assignments based on the literature^15^.

Wavenumber (cm^−1^)	Vibrational mode	Phase assignment
878	P-O stretching mode of PO_4_ group	Brushite
948	P-OH stretching mode of HPO_4_^2−^	TCP
960	P-O stretching mode of PO_4_ group	OCP
970	P-O stretching mode of PO_4_ group	TCP
985	P-O stretching mode of PO_4_ group	Brushite
1010	P-O stretching mode of HPO_4_^2−^	OCP

**Table 2:** Quantification of area and penetration changes for phases at each time point for samples aged in PBS based on the dominant phase calculated at each spatial location.

Time (days)	Composition (wt%)			Approximate OCP penetration (μm)
	Brushite	OCP	TCP	
0	89	0	11	0
5	88	1	11	150
10	90	6	4	350
20	50	8	42	520
30	38	23	39	750
50	13	44	43	1300
